# Gender disparity in health-related quality of life and fatigue after living renal donation

**DOI:** 10.1186/s12882-018-1187-8

**Published:** 2018-12-27

**Authors:** Claudia Sommerer, Sarah Estelmann, Nicole G. Metzendorf, Maren Leuschner, Martin Zeier

**Affiliations:** 1 Division of Nephrology, Medical University Hospital Heidelberg, Im Neuenheimer Feld 162, 69120 Heidelberg, Germany; 2 Department of General Internal and Psychosomatic Medicine, University Hospital Heidelberg, Im Neuenheimer Feld 410, D-69120 Heidelberg, Germany

**Keywords:** Living renal donation, Gender, Quality of life, Fatigue, Depression

## Abstract

**Background:**

The clinical outcome and health-related quality of life (HRQoL) of living kidney donors is mostly not detrimental, but some donors experience impairment after donation. Gender-specific effects of living kidney donors was evaluated.

**Methods:**

Clinical outcome was assessed in living kidney donors and HRQoL was obtained by self-reporting validated test systems as the Multidimensional Fatigue Inventory (MFI-20), the Short Form 36 (SF-36), and the Patient Health Questionnaire (PHQ-9).

**Results:**

Two hundred and eleven (211) living renal donors were evaluated (female 62.2%). Response rate was 80.8%. In both genders, a decrease of renal function of 26% was observed after donation. De novo antihypertensives were introduced in 28.3% of women and 36.5% of men. HRQoL was comparable in female and male donors, except for mental HRQoL, which was lower in 51- to 60-year-old female donors, compared to age-matched male donors and to the female general population. Female donors aged 40–59 years demonstrated more fatigue than the age-matched general population. A low mental HRQoL (MCS; SF-36) was associated with higher values for fatigue (General Fatigue Score; MFI-20) in both genders. Multiple regression analysis detected the General Fatigue score of the MFI-20 questionnaire and depression identified by the PHQ-9 score as independent variables predicting MCS of the SF-36 in both genders. Lower age at time of donation contributed to a lower MCS in female donors.

**Conclusions:**

Overall, HRQoL in living kidney donors exceeds that of the general population. Inferior mental health status and fatigue seem to be a problem, especially in middle-aged female donors, but not in all female donors. Psychological evaluation pre donation and psychological support post donation are required.

**Electronic supplementary material:**

The online version of this article (10.1186/s12882-018-1187-8) contains supplementary material, which is available to authorized users.

## Background

The number of living renal donations is increasing worldwide [1]. Altogether, 1921 renal transplantations were performed in Germany in 2017, of which 557 (29%) kidneys were from a living donor [2]. In all the cases, a healthy person decided to undergo the risks of surgery mostly for altruistic reasons [3]. Therefore, living donation has a special status from the medical and ethical points of view. However, there are studies that hint at the presence of fatigue and other psychosocial problems post donation, particularly for the subgroup of female donors and maybe also for a special age category [4, 5]. Gender medicine in living renal donation is of high importance: there are more women donating an organ than men, and women are less often given a kidney by a living donor [6]. Another point is the difference between male and female living donors concerning clinical outcome: a study by Mjoen et al. showed that female donors with a BMI > 25 kg/m^2^ and an age > 50 years achieved the lowest GFR after donation [7].

Altogether, clinical outcome and HRQoL of living kidney donors mostly seem to be safe, but some donors experience impairment after donation. In the present study, to detect donors at risk, female and male kidney donors are evaluated concerning physical and psychosocial outcomes.

## Methods

### Study population

The study was designed to assess clinical and psychosocial outcome in living renal donors with respect to gender differences. Two hundred and ninety-three (293) kidney donors were contacted by mail, including an invitation letter as well as standardised questionnaires. All these participating respondents donated a kidney between 08/1983 and 06/2011.

HRQoL assessment and screening for fatigue or depression was determined by self-reporting validated test. A representative German adult general population cohort served as the control group for each questionnaire. A regular clinical follow-up after donation was performed annually. Psychological counselling was undertaken for each donor and recipient prior to transplantation and, if necessary, after transplantation.

The study was approved by the institutional Ethics Committee and was conducted according to the Declaration of Helsinki 2003. Written informed consent was obtained from all participating donors.

### Questionnaires

#### Short form 36

The Short Form 36 (SF-36) is a questionnaire to measure HRQoL containing the eight multi-item subscales: general health perceptions, physical functioning, physical role, bodily pain, general mental health, vitality, emotional role, and social functioning. Each subscale has a range from 0 to 100 with 100 standing for optimal function. The subscales are then combined into a physical and mental component summary score (PCS and MCS). We compared the study population with the German general population presented by the study of Ellert and Bellach et al. (6964 persons between the age of 8 and 80 years) [8, 9].

#### Multidimensional fatigue inventory

The Multidimensional Fatigue Inventory (MFI-20) was designed to measure chronic tiredness and was originally conceived for patients with fatigue due to malignoma. There are 20 items, each consisting of a 5-point Likert Scale. The 20 items are built on five summary scales: general fatigue, physical fatigue, mental fatigue, reduced activity and reduced motivation. A higher score indicates more fatigue. We compared our study population to the German general population of Schwarz et al. (2037 persons) [10, 11].

#### Patient health questionnaire - depression component questionnaire

The Patient Health Questionnaire (PHQ) was designed to detect mental disorders. In our study we used the “depression” component questionnaire of the PHQ (PHQ-9). The questionnaire consists of 9 Items, each consisting of a 4-point Likert Scale. The questions include a period of the last two weeks. A summary score can be build with the following cut-off scores: 0–4 points indicates no mental disorder, 5–10 points can be seen as a probable beginning mental disorder. 11–14 points indicate a mild, 15–19 a distinctive and 20–27 a severe form of major depression [12]. We compared our donors with a normal sample of Kocalevent et al. (2013) [13].

#### Statistical analysis

All analyses were performed using IBM SPSS Statistics for Windows, Version 21.0 (IBM Corp. released 2012; Armonk NY: IBM Corp.). Values are presented as mean (SD) or n (%).

Statistical significance was tested with paired and unpaired t-tests, as well as chi-square distribution. *p* < 0.05 indicated statistical significance. Multiple linear regression analyses was performed, using the “Mental Component Summary” of the SF-36 as the dependent variable, and General Fatigue Score, Physical Component Summary Score, PHQ-9, S-creatinine, age at time of donation and time after donation as independent variables.

## Results

### Study population

**Fig. 1:**
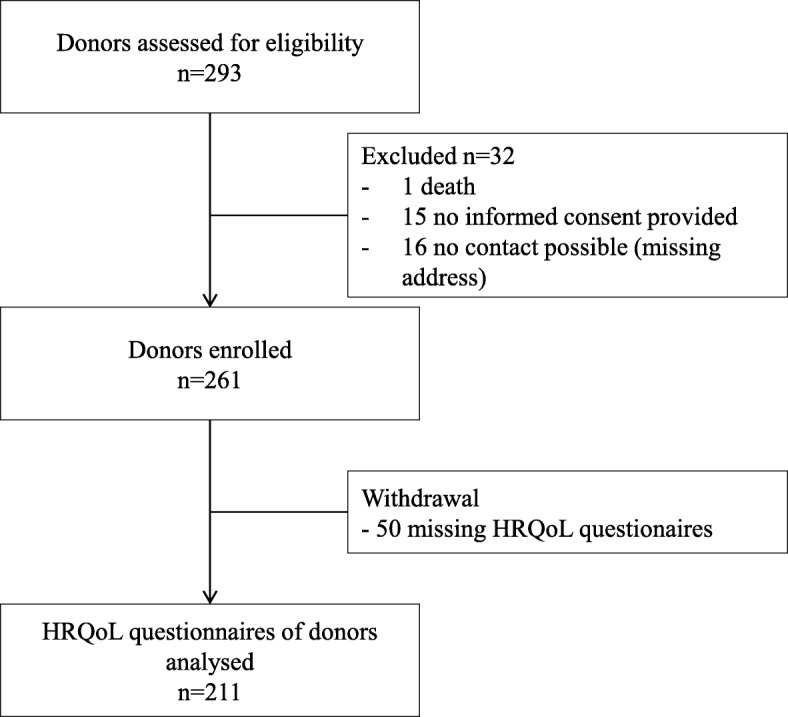
Donor flow chart

A total of 293 living renal donors were contacted. Out of 261 expected responses, 211 questionnaires were returned by donors (80.8%), Fig. 1. Altogether, 131 (62.1%) of the 211 participating renal donors were female. The return rates, separated by gender, were 76.9% in male and 69.7% in female donors (*p* = 0.165). Mean age at the time of donation was 51.7 ± 9.9 years (female 50.5 ± 9.3 years; male 53.8 ± 10.6 years) and, at time of the assessment, 61.5 ± 10.2 years (female 60.4 ± 9.6 years; male 63.2 ± 10.9 years), Table 1. Most of the donors were spouses (26.1%), mothers (24.6%) or fathers (22.7%). Male donors were more likely to donate to their children (60%), whereas female donors were willing to donate to their children (39.7%) as well as to their husbands (42.7%).

**Table 1:** Patient demographics separated by gender

	All donors *N* = 211	Female donors *N* = 131	Male donors *N* = 80	*p*
Gender, *n* (%)	–	131 (62.1)	80 (37.9)	–
Age at the time of donation (years), mean (SD)	51.7 (9.9)	50.5 (9.3)	53.8 (10.6)	0.057
Age at the time of assessment (years), mean (SD)	61.5 (10.2)	60.4 (9.6)	63.2 (10.9)	0.019
Time after donation (years), mean (SD)	9.7 (5.2)	9.9 (5.4)	9.4 (4.8)	0.459
Relationship to recipient, *n* (%)				
Parent (father/mother)	100 (47.4)	52 (39.7)	48 (60)	0.004
Child (son/daughter)	2 (0.9)	–	2 (2.5)	
Sibling (brother/sister)	28 (13.3)	19 (14.5)	9 (11.3)	
Spouse (husband/wife)	71 (33.6)	56 (42.7)	15 (18.8)	
Friends, emotionally related	3 (1.4)	1 (0.8)	2 (2.5)	
Other related	7 (3.3)	3 (2.3)	4 (5.0)	
Children mean (SD)	2.2 (0.90)	2.2 (1.0)	2.1 (0.7)	0.375
Smoking, *n* (%)				
Smoker	34 (16.1)	23 (17.6)	11 (13.8)	
Non-smoker	174 (82.5)	107 (81.7)	67 (83.8)	
Unknown	3 (1.4)	1 (0.8)	2 (2.5)	
Donor medical prevention, *n* (%)				
Yes, routinely	136 (64.5)	89 (67.9)	47 (58.8)	0.176
No	28 (13.3)	11 (8.4)	17 (21.3)	
Occasionally	47 (22.3)	31 (23.7)	16 (20.0)	

*N* number, *p* significance, *SD* standard deviation

Demographic data of donors with missing HRQoL questionnaires (69.5% female) were comparable to the studied donor cohort with a mean age of 58.4 ± 9.3 years and stable renal function.

### Clinical data

Comparing pre and post donation parameters, S-creatinine was significantly higher after donation (S-creatinine 0.82 ± 0.16 vs. 1.05 ± 0.23 mg/dL, *p* > 0.001), Table 2. S-creatinine differed significantly in female and male donors before and after donation (pre 0.74 ± 0.12 mg/dL vs. 0.94 ± 0.14 mg/dl, *p* < 0.001; post 0.90 ± 0.15 vs. 1.20 ± 0.22 mg/dL, *p* < 0.001). Estimated GFR was comparable between female and male donors before and after donation, but decreased significantly after donation (*p* < 0.001). Decrease of CKDepi GFR was 24 ml/min in female and 23 ml/min in male donors. Proteinuria increased after donation in both gender with significantly higher post donation proteinuria in male compared to female donors. However, the general amount of proteinuria was low.

**Table 2:** Clinical parameters of donors separated by gender after living renal donation

	Female	Male	Significance
	Mean (SD)	Mean (SD)	*p*
S-creatinine [mg/dL] - pre	0.74 (0.12)	0.94 (0.14)	< 0.001
S-creatinine [mg/dL] – post	0.90 (0.15)	1.22 (0.22)	< 0.001
Significance *p*	< 0.001	< 0.001	
CKD-EPI GFR [mL/min/1.73m^2^] - pre	93 (14)	91 (14)	0.393
CKD-EPI GFR [mL/min/1.73m^2^] – post	69 (15)	68 (15)	0.591
Significance *p*	< 0.001	< 0.001	
Proteinuria [g/L] - pre	0.04 (0.03)	0.05 (0.08)	0.307
Proteinuria [g/L] – post	0.05 (0.04)	0.08 (0.11)	0.028
Significance *p*	< 0.001	< 0.001	
BMI [kg/m^2^] - pre	25.3 (4.7)	26.2 (3.5)	0.133
BMI [kg/m^2^] – post	26.4 (4.9)	27.4 (3.5)	0.307
Significance *p*	0.179	0.058	
Blood pressure- systolic [mmHg] - pre	127 (14)	133 (12)	0.001
Blood pressure- systolic [mmHg] – post	131 (15)	132 (15)	0.662
Significance *p*	0.006	0.568	
Blood pressure-diastolic[mmHg] - pre	80 (8)	83 (8)	0.007
Blood pressure-diastolic[mmHg] – post	82 (8)	81 (7)	0.257
Significance *p*	0.006	0.025	
Cholesterol [mg/dL] - pre	214 (39)	213 (38)	0.830
Cholesterol [mg/dL] – post	214 (46)	207 (41)	0.373
Significance *p*	0.266	0.276	
HbA1c [mg/dL] - pre	5.5 (0.5)	5.6 (0.4)	0.239
HbA1c [mg/dL] – post	5.5 (0.4)	5.5 (0.4)	0.912
Significance *p*	0.186	0.291	

BMI body *mass index,* CKD-EPI chronic kidney disease e*pidemiological,* GFR glomerular filtratio*n rate,* HbA1c ha*emoglobin A1c,* N *number,* p *significance,* S serum*,* SD standard deviation

Mean systolic blood pressure was 131 ± 15 mmHg and diastolic blood pressure was 82 ± 7 mmHg prior to donation. Most females demonstrated a blood pressure in the category of high normal or stage 1 hypertension (both 30.6%). Most males also showed high normal values (31.0%). Especially in female donors, blood pressure increased significantly after donation. However, percentages of de novo prescription of antihypertensives after donation was 28.3% in female and 36.5% in male.

Body mass index (BMI), cholesterol and HbA1c did not differ significantly in female or male donors prior and post donation, Table 2.

### Health-related quality of life (SF-36)

Female and male donors showed comparable results for PCS (Physical Component Summary Score, 51.8 ± 10.1 vs. 53.9 ± 7.9). Significantly worse results were achieved by female compared to male donors in MCS (Mental Component Summary Score, 47.3 ± 13.0 vs. 51.7 ± 11.0, *p* = 0.012), Table 3. The best results were achieved in the scale of “Social Functioning” by male living donors, whereas female living donors achieved the best results in the scale of “Physical Functioning”.

**Table 3:** Short Form 36 (SF-36): Mean (SD) of the subscales and physical and mental component summary score in living renal donors compared to the German general population of 1998 separated by gender [11]

	Female			Male		
	Mean (SD) female donors	Mean female German reference (1998)	*p*	Mean (SD) male donors	Mean male German reference (1998)	*p*
PF	82.70 (21.84)	82.77	0.972	88.73 (14.15)	88.18	0.729
RP	78.46 (34.83)	79.22	0.804	88.46 (26.02)	85.53	0.323
BP	74.35 (28,34)	63.89	< 0.001	82.43 (23.74)	71.04	< 0.001
GH	69.77 (18.37)	66.03	0.021	70.22 (18.07)	66.83	0.102
VT	59.78 (19.73)	57.57	0.201	67.03 (17.82)	62.58	0.030
SF	81.06 (25.49)	84.24	0.157	90.94 (16.15)	88.63	0.205
RF	80.77 (35.43)	86.74	0.057	87.61 (28.98)	91.58	0.230
MH	70.14 (19.80)	69.83	0.857	76.56 (17.72)	75.22	0.505
PCS	51.79 (10.05)	47.49	< 0.001	53.89 (7.94)	49.26	< 0.001
MCS	47.26 (12.96)	49.85	0.026	51.71 (11.0)	51.92	0.868

*PF* Physical Functioning, *RP* Role Percept-ion, *BP* Bodily Pain, *GH* General Health Percept-ion, *VT* Vitality, *SF* Social Func-tioning, *RF* Role Functioning, *MH* Mental Health, *PCS* Physical Component Summary Score; MCS, Mental Component Summary Score; p, significance; SD, standard deviation

In comparison to the German general population, living renal donors showed significantly higher results in PCS (52.6 ± 9.3 vs. 48.36 ± 9.42; *p* < 0.001) and significantly lower results in MCS (48.7 ± 19.5 vs. 50.78 ± 8.82; *p* = 0.037). Lower MCS results in the donor cohort were induced due to significantly lower MCS in female donors compared to females in the German general study population, whereas male revealed no difference.

Further analysis revealed that the female donor group aged 51 to 60 years contributed to this phenomenon with lower results in MCS (41.4 ± 14.6 vs. 50.1 ± 9.6, *p* < 0.001) and in the subscale of “Mental Health” (65.4 ± 41.6 vs. 84.2 ± 30.7, *p* = 0.002) in comparison to the age- and gender-matched German general population. All other age groups in all sub- and summary scales showed comparable or even better results. Also in comparison to male donors, only female donors aged 51–60 years revealed significantly worse results in MCS (41.6 ± 14.7 vs. 51.5 ± 8.9, *p* = 0.008). Male donors showed comparable or even significantly better results in PCS and MCS, compared to the age- and gender-matched German general population.

### Fatigue (MFI-20)

Comparing female and male donors, a significant difference was detected in the scale of “General Fatigue” with women showing higher, i.e. worse, results than men (9.9 ± 4.7 vs. 8.4 ± 4.0, *p* = 0.013). Especially female donors aged 40–59 years showed significantly higher results in the scales of “General Fatigue” (11.2 ± 4.7 vs. 8.7, *p* < 0.001) and “Physical Fatigue” (9.7 ± 4.5 vs. 8.2, *p* = 0.014) than the age- and gender-matched German general population, Table 4. The age group of > 60-year-old female as well as male donors showed significantly lower results in almost all the fatigue scales.

**Table 4:** Multidimensional Fatigue Inventory (MFI-20): Mean (SD) of the MFI-20 scales in living renal donors compared to the German general population separated by gender and age [15]

	Female			Male		
	Age ≤39^a^	Age 40–59	Age ≥ 60	Age ≤39^a^	Age 40–59	Age ≥ 60
General Fatigue						
N	3	60	68	1	29	48
Mean	7.67	11.16	8.96	17.0	9.21	7.65
SD	2.52	4.74	4.43	–	4.09	3.70
German reference (Mean)	7.7	8.7	10.8	6.6	8.0	10.1
*p*	–	< 0.001	0.001	–	0.123	< 0.001
Physical Fatigue						
N	3	59	68	1	29	50
Mean	8.0	9.7	7.91	11.0	8.08	7.5
SD	4.58	4.52	3.36	–	3.74	3.26
German reference (Mean)	6.8	8.2	11.1	6.1	7.6	10.3
*p*	–	0.014	< 0.001	–	0.495	< 0.001
Reduced Activity						
N	3	60	68	1	29	50
Mean	6.33	9.09	7.63	15.0	7.91	7.66
SD	2.08	4.37	3.48	–	3.9	3.54
German reference (Mean)	7.1	8.2	10.5	6.4	7.6	10.3
*p*	–	0.121	< 0.001	–	0.674	< 0.001
Reduced Motivation						
N	3	60	68	1	29	50
Mean	6.67	7.68	7.16	17.0	8.07	7.26
SD	3.79	3.54	3.28	–	4.41	3.15
German reference (Mean)	6.7	8.0	9.9	6.2	7.6	9.1
Significance	–	0.484	< 0.001	–	0.571	< 0.001
Mental Fatigue						
N	3	60	68	1	29	50
Mean	7.67	7.82	7.57	19.0	8.34	7.9
SD	4.73	4.17	3.54	–	3.65	3.11
German reference (Mean)	7.1	7.8	9.2	6.4	7.1	8.7
*p*	–	0.967	< 0.001	–	0.077	0.075

^a^No analysis of this group because of too small number of cases

*p* significance, *SD* standard deviation

### Depression (PHQ-9)

Male and female donors presented a mean value of 3.86 ± 4.15, which is far under the cut-off score of 10 points for depressive disorders (male: 3.24 ± 3.48, female: 4.26 ± 4.50; *p* = 0.087), Table 5. Only 6.3% of the male and 10.5% of the female donors presented PHQ-9 values above 10 points. In comparison to the German normal population (*N* = 5018) the donor cohort showed a significantly higher summary score (2.91 ± 3.52 vs. 3.86 ± 4.15, *p* = 0.001). Separated by gender and age, the male as well as the female donors showed comparable or even better (> 75 year old male and females: 3.1 ± 3.2 vs. 4.4 ± 3.9, *p* = 0.041) results than the age- and gender- matched German normal sample.

**Table 5:** PHQ-9 “Depression”: Comparison of living renal donors (a. female, b. male) with the German general population of Kocalevent et al. (2013) in age classes [13]

Age categories	Mean (SD)	N	Mean (SD)	N	Sign^a^
a					
	Female donors		Women of the German normal population		
25–34 years	1.0 (−)	1	2.5 (3.0)	351	–
35–44 years	4.3 (3.6)	6	2.8 (3.6)	542	–
45–54 years	3.7 (3.8)	26	2.7 (3.3)	457	0.174
55–64 years	4.4 (4.9)	43	3.3 (3.4)	446	0.125
65–74 years	4.8 (5.1)	39	3.6 (3.6)	395	0.163
≥75 years	3.0 (2.2)	9	4.5 (3.5)	236	0.076
b					
	Male donors		Men of the German normal population		
25–34 years	1.0 (−)	1	2.0 (3.2)	279	–
35–44 years	4.3 (6.0)	4	2.3 (3.3)	396	–
45–54 years	4.1 (3.7)	18	2.9 (3.7)	414	0.174
55–64 years	2.7 (3.3)	22	3.1 (3.6)	398	0.584
65–74 years	2.9 (2.8)	18	3.0 (3.6)	397	0.855
≥75 years	3.1 (3.6)	18	4.1 (4.4)	156	0.266

^a^unpaired T-Test

### Multiple regression analyses

Multiple regression analysis identified fatigue symptoms detected by the General Fatigue score of the MFI-20 questionnaire and depression perceived by the PHQ-9 score as independent variables predicting MCS of the SF-36 in both genders (Table 6). Lower age at time of donation supported a lower MCS in female donors.

**Table 6:** Multiple Regression Analysis. Presenting regression coefficient β, standard error and significance p of the presented independent variables on the dependent variable “Mental Component Summary Score” of the Short-Form 36, separated by gender

	Female Donors			Male Donors		
	Regression Coefficient β	Standard error	*p*	Regression Coefficient β	Standard error	*p*
(constant)	71.812	11.209	0.000	82.210	15.308	0.000
Age at time of donation	0.178	0.077	0.023	0.055	0.078	0.487
Mean arterial pressure post donation	−0.127	0.076	0.100	−0.062	0.107	0.568
Renal function (S-creatinine mg/dL) post donation	1.981	2.224	0.375	−1.050	3.759	0.781
Recipient outcome	−0.451	0.417	0.282	0.125	0.594	0.834
Physical Component Summary Score	−0.066	0.082	0.424	−0.192	0.119	0.112
General Fatigue	−1.347	0.220	0.000	−1.249	0.299	0.000
PHQ-9	−1.445	0.255	0.000	−1.461	0.306	0.105

*MCS* Mental Component Summary Score, *PCS* Physical Component Summary Score, *S* serum, PHQ-9 Depression Patient Health Questionnaire

### Characteristics of middle-aged female donors with impaired quality of life

To further analyse impaired quality of life in female donors aged 51–60 years we evaluated female donors of this age-group with results lower than two standard deviations from the mean value of the age and gender matched German general population in the MCS of the SF-36 (*n* = 15) and compared them with female donors above this value, Additional file 1: Table S1.

No significant difference could be detected in renal function of the donor (S-creatinine, *p* = 0.227), renal function of recipient (S-creatinine, *p* = 0.961) or loss of kidney transplant of the recipient (*p* = 1.000). Female donors who showed very low results in MCS also showed significantly higher values for fatigue (*p* < 0.001) in comparison to female donors with normal value in MCS. These women presented very low results in the subscale “emotional role functioning” of the Short-Form 36 in comparison to the age- and gender-matched normal population (15.56 ± 21.33 vs. 84.16 ± 30.70, *p* = 0.002).

Most of theses 15 women donated the kidney to their husband (*N* = 7), followed by donating to their child (*N* = 6), the others donated to their brother/sister (*N* = 2); 53.3% (*n* = 8) were working in a part time job, 3 in a full time job (others: retired *n* = 1, unemployed n = 1, housewife *n* = 2). These women had 2.08 children (range 1–3). Mean age at time of donation was 45.1 ± 7.7 years. Mean GFR was 73 ± 17 ml/min/1.73m^2^ and S-creatinine was 0.88 ± 0.13 mg/dL. Mean arterial pressure was 99 ± 9 mmHg at the time of donation.

## Discussion

Altogether, 211 living renal donors were evaluated concerning clinical and psychosocial outcome. There was no difference in terms of gender concerning the renal function at about ten years post donation, whereas S-creatinine was significantly higher in the males. The psychosocial evaluation of the donor cohort revealed better results in HRQoL in male and female donors, expect for the patient group of 51- to 60-year-old female donors. Evaluation of fatigue by the MFI-20 questionnaire demonstrated comparable results for both genders, except for the scale of “General Fatigue” in female donors aged 40 to 59 years who obtained worse results than male donors.

The response rate of 80.8% was comparable to other psychosocial outcome evaluations in renal donors [14, 15]. The mean age of the living female donors in this study was 60.4 ± 9.6 years and the mean age of male donors was 63.2 ± 10.9 years. Overall, 55.9% of the donors were aged > 60 years. Dols et al. could not show a significant difference in the decline of GFR, in a comparison between donors < 60 years and donors > 60 years, with older donors already demonstrating a lower GFR prior to donation [16]. However, in this study no disadvantage due to increased age could be shown.

Concerning blood pressure, there were no significant differences between men and women, or between time pre and post donation. However, 28.3% of the females and 36.5% of the males obtained de novo antihypertensive drugs after donation. Fehrman-Ekholm et al. demonstrated comparable results: 23% of the donors needed de novo antihypertensive drugs after donation, and 22% demonstrated a newly diagnosed arterial hypertension [17]. Nevertheless, the prevalence of hypertension in the German general population is similar, at 29.9% in females and 33.3% in males [18].

Renal function was estimated by the CKD-EPI formula. This formula provides the most precise results in the Normal to Stage CKD II categories [19]. No patient in this study showed an eGFR below 30 mL/min/1.73m^2^. Corresponding to previous evaluations, a mean decrease of 26% was noted after donation [17, 20]. eGFR and change in renal function were equivalent in both genders. In a study by Ibrahim et al., a mean eGFR decrease of 24% was noted [21]. A better compensation of the GFR was possible at a younger age, longer time since donation and a higher estimated GFR at the time of donation. Mjoen et al. revealed women aged > 50 years with a BMI > 25 kg/m^2^ as being at high risk for decreased renal function [7]. In a most recent study, GFR decreased about 33.6% and was longitudinally lower among men than women, without any association to HRQoL [22].

Physical and mental HRQoL assessed by the SF-36 questionnaire was significantly better in male living donors in total and in different age groups, than in males of the German general population [9]. Female donors in this study showed significantly better physical HRQoL, in comparison to the female general population, but reduced mental health in comparison to the general population. Women aged 51–60 years in particular achieved lower results in mental health. On the contrary, Ibrahim et al. showed better results for both genders in comparison to the US general population [21]. In a recently published study, donors felt positive about donation and there was no evidence of a significant change in psychosocial outcomes [23]. Timmerman et al. evaluated the importance of psychological factors on mental health after living renal donation [24]. Lower age, lack of social support, expectation of interpersonal benefit, an avoidant coping style were identified as indicators of a lower mental health status. Kroencke et al. demonstrated that the donor-recipient relationship might influence HRQoL outcome in living renal donation. Adult-to-paediatric donors experienced more preoperative psychological stress, which improved after donation, whereas adult-to-adult donors showed unchanged anxiety and depression, and a slight decrease in mental health [25]. Female donors of our study, who had results below two standard deviations in MCS of the Short-Form 36 donated mostly to a child as well as to their husband.

Important results were found in the present study: low results in MCS of the SF-36 came along with higher values for Fatigue (MFI-20). These findings are in line with de Groot et al., who identified higher fatigue in all dimensions of the MFI-20 in donors (female and male) with reduced PCS and MCS subscales [15].

Fatigue is not easily measured objectively and mostly set as a diagnosis by exclusion. A lot of differential diagnoses, such as somatoform disorder or depression have to be considered. Demyttenaere et al. showed fatigue as a common symptom of a major depression, whereas Artom et al. showed fatigue in advanced kidney diseases as a diagnosis in itself [26, 27]. In a Norwegian analysis female sex in general was significantly associated with general fatigue [28]. In our study women aged 40 to 59 years showed significantly worse results in the “General Fatigue” scale and the “Physical Fatigue” scale, whereas women aged > 60 years showed significantly better results than the German general female population. In contrast to other evaluations, a linear correlation between age and fatigue therefore could not be noted in the present study [11, 29].

Female donors especially of the age group of 51–60 years seem to be at a higher risk for low mental health after living renal donation, although the reason for that fact remains unclear. Our results show that the outcome of recipients, measured by S-creatinine, did not correlate with a bad mental health of the donors. Lopez et al. showed a significant correlation between low resilience, depressive symptoms and being in peri-menopause [30]. Perhaps women at the age of 51–60 years have to deal with a lot of stressors e.g. changing of the body in menopause, children who become adults and maybe own parents who have to be cared about. Further analyses of this special age group should be performed in following studies.

The benefit of the present study is that it provides a comprehensive evaluation including physical, mental and social outcome of all living renal donors at the Transplant Centre of Heidelberg. Limitations of this study include the cross-sectional evaluation of HRQoL, whereas the clinical follow-up was assessed prospectively in yearly follow-up visits. Nearly 19.8% of the kidney donors refused to complete the self-reported questionnaires. However, demographics of these donors were similar to the assessed donors. There is no consent among experts as to the best control group. Comparing living donors to national data may underestimate the psychosocial impairment attributable to kidney donation.

## Conclusions

In conclusion, no significant difference between female and male donors concerning physical outcome could be detected. HRQoL is mostly comparable, or even better, in kidney donors compared to the general population. Limited HRQoL and fatigue symptoms seem to be a problem especially in middle-aged female donors, but not in all female donors. This special patient cohort could be identified by low mental health or high fatigue. Therefore, we suggest psychological counselling as part of the evaluation of potential living renal donors as well as part of the after-care programme.

## Additional file

Additional file 1 **Table S1.** Comparison of the female donors of the age group 51–60 years with results lower than two standard deviations from the mean value of the age and gender matched German general population in the “Mental Component Summary Score” of the SF-36 with female donors above this value. (DOCX 16 kb)

## Abbreviations

BMI: Body mass index
CKD-EPI: Chronic kidney disease epidemiological formula
eGFR: Estimated glomerular filtration rate
GFR: Glomerular filtration rate
HADS-D: Hospital Anxiety Depression Scale (German version)
HRQoL: Health-related quality of life
MCS: Mental component summary
MFI-20: Multidimensional Fatigue Inventory
p: significance
PCS: Psychological component summary
SD: Standard deviation
SF-36: Short Form 36
WHO: World Health Organisation
