# Supplementary material for: Gender disparity in health-related quality of life and fatigue after living renal donation
Source: BMC Nephrol. 2018 Dec 27;19:377. doi: 10.1186/s12882-018-1187-8 (PMC6307222; doi:10.1186/s12882-018-1187-8)
Supplement: Supplementary file 1 — Table S1. Comparison of the female donors of the age group 51–60 years with results lower than two standard deviations from the mean value of the age and gender matched German general population in the “Mental Component Summary Score” of the SF-36 with female donors above this value. (DOCX 16 kb) [file 12882_2018_1187_MOESM1_ESM.docx]

**Additional File 1**

**Table S1**

Comparison of the female donors of the age group 51-60 years with results lower than two standard deviations from the mean value of the age and gender matched German general population in the “Mental Component Summary Score” of the SF-36 with female donors above this value.

|  | Women aged 51-60 years ≤2SD in MCS | All other women of the age group 51-60 years |  |
| --- | --- | --- | --- |
|  | Mean (SD)  N=15 | Mean (SD)  N=37 | Significance |
| Age at donation (years) | 45.1 (6.7) | 47.5 (5.5) | 0.186 |
| Time since donation (years) | 9.7 (6.3) | 8.7 (4.3) | 0.525 |
| Renal function of the donor  (S-creatinine, mg/dL) | 0.88 (0.13) | 0.93 (0.11) | 0.227^a^ |
| Renal function of recipient  (S-creatinine, mg/dL) | 2.20 (3.56) | 2.15 (2.89) | 0.961^a^ |
| Loss of kidney transplant of the recipient (absolute number) | 2 | 6 | 1.000^b^ |
| General Fatigue Scale (MFI-20) | 16.2 (3.3) | 9.6 (4.2) | 0.000^a^ |
| Depression (PHQ-9) | 9.0 (2.4) | 3.2 (3.0) | 0.000^a^ |
| Physical component score (SF-36) | 44.4 (11.2) | 52.4 (10.5) | 0.017^a^ |

^a^independent samples T-Test; ^b^Fisher`s exact test for count data
